# Tuberculosis caseload in children with severe acute malnutrition related with high hospital based mortality in Lusaka, Zambia

**DOI:** 10.1186/s13104-017-2529-5

**Published:** 2017-06-12

**Authors:** Tendai Munthali, Chishala Chabala, Elson Chama, Raider Mugode, Nathan Kapata, Patrick Musonda, Charles Michelo

**Affiliations:** 1 School of Public Health, University of Zambia, P.O Box 50110, Lusaka, Zambia; 2 Ministry of Health, P.O Box 30205, Lusaka, Zambia; 3 Department of Paediatrics and Child Health, University Teaching Hospital, Lusaka, Zambia; 4 School of Medicine, University of Zambia, Lusaka, Zambia; 5 National Food and Nutrition Commission, Lusaka, Zambia

**Keywords:** TB, Acute-malnutrition, Mortality, Hospital-based

## Abstract

**Background:**

Tuberculosis and severe acute malnutrition (SAM) in children pose a major treatment and care challenge in high HIV burden countries in Africa. We investigated the prevalence of Tuberculosis notifications among hospitalised under-five children with severe acute malnutrition. A retrospective review of medical records for all children aged 0–59 months admitted to the University Teaching Hospital from 2009 to 2013 was performed. Descriptive statistics were employed to estimate TB caseload. Logistic regression was used to identify predictors of the TB caseload.

**Results:**

A total of (n = 9540) under-five children with SAM were admitted over the period reviewed. The median age was 16 months (IQR 11–24) and the proportion diagnosed with TB was 1.58% (95% CI 1.3, 1.8) representing 151 cases. Of these, only 37 (25%) were bacteriologically confirmed cases. The HIV seroprevalence of children with SAM and TB was 46.5%. Children with SAM and TB were 40% more likely to die than children with SAM and without TB.

**Conclusions:**

Tuberculosis contributes to mortality among children with SAM in high TB and HIV prevalence settings. The under detection of cases and association of TB with HIV infection in malnutrition opens up opportunities to innovate integrative case finding approaches beyond just HIV counselling and testing within existing mother and child health service areas to include TB screening and prevention interventions, as these are critical primary care elements.

**Electronic supplementary material:**

The online version of this article (doi:10.1186/s13104-017-2529-5) contains supplementary material, which is available to authorized users.

## Background

Tuberculosis (TB) and malnutrition are important causes of morbidity and mortality in children in the developing world [1, 2]. In 2015, an estimated 1 million children developed TB and 170,000 died from it [2]. Further, an estimated 50 million children suffer from severe wasting worldwide resulting in nearly 1 million deaths annually mostly in Sub-Saharan Africa and Asia [3, 4]. Malnutrition increases the risk of developing TB and is also a consequence of TB [5, 6].

Severe acute malnutrition is associated with serious lower respiratory tract infections including TB and pneumonia and facilitates the rapid progression of TB infection to active disease due its immunosuppressive effect [7–9]. This effect is compounded by HIV infection and other childhood illnesses that increase the risk of TB.

Both TB and malnutrition are common public health problems in Zambia. The TB prevalence of 455/100,000 is among the highest in Africa and the country was recently classified by the World Health Organisation (WHO) as one of the 30 high TB burden countries in the world [10, 11]. Children account for <10% of incident TB cases annually [12]. The adult HIV infection is 12.3% and has largely contributed to an exponential rise in TB over the last 3 decades [13]. Among children, more 50% of TB patients are HIV co-infected [14, 15]. SAM is endemic in Zambia affecting 5% in 2007 to 6% in 2014 of under-five children [16–18].

Despite the strong epidemiological association between malnutrition, TB and HIV infection in most sub-Saharan African countries, the burden of TB among severely malnourished children is not well defined. The aim of this study was to determine the burden of TB and its contribution to mortality in children with SAM at the largest referral hospital in Zambia.

## Methods

### Study setting and participants

A retrospective, cross-sectional study of patient files, ward and discharge registers for all children aged 0–59 months admitted to the paediatric nutritional rehabilitation unit at the University Teaching Hospital (UTH) in Zambia from 2009 to 2013 was performed. The UTH nutritional unit is the main referral centre for children with complicated malnutrition. It has a limited bed capacity of 59 cots but admits about 1500–2000 cases annually which often results in overcrowding of patients. The ward is managed by rotating resident registrars, junior resident medical officers, a senior registrar, a consultant paediatrician and three to five nurses per shift. All admissions are examined by the attending physician and co-morbidities were assessed clinically and appropriate investigations ordered as indicated. Admission to the unit is based on the presence of bilateral pitting oedema and/or weight for height Z-scores (WHZ) <−3 standard deviations (SD). The WHO weight-for-length reference card (growth standards charts) are used to calculate weight for height Z-scores [5].

### Clinical care and procedures

Tuberculosis was diagnosed based on any of the following: clinical evaluation (with or without TB contact history), suggestive radiological findings and/or positive ZN smear results of gastric aspirates. At the time of this review, mycobacterium cultures, tuberculin skin test and the Xpert MTB-RIF test were not routinely available. According to unit practice; all severely malnourished children were expected to have three early morning gastric aspirates performed but due to logistical and staff constraints this is only performed on children in whom the attending physicians have a strong clinical suspicion. Children diagnosed with TB are notified and managed in accordance with the Zambia National Childhood Tuberculosis Treatment Guidelines. HIV screening for all children with SAM was done using Determine^®^HIV-1/2 test. HIV seropositive children younger than 18 months have HIV DNA PCR performed to confirm HIV infection and those with confirmed infection on ART in accordance with Zambia Paediatric HIV treatment guidelines [19]. Management of SAM is based on the WHO standard guidelines for the therapeutic management of SAM [20]. A two phased approach using F75 therapeutic milk in the first phase (prepared using fresh or fermented milk on the ward) and a second phase that uses F100 therapeutic feed or plumpy nut-based ready-to-use therapeutic feed (RUTF). Children successfully treated are discharged and referred to one of the outpatient therapeutic programs (OTP) within the city for full recovery.

### Data extraction and analysis

Trained assistants extracted data on variables of interest from patient ward and discharge registers and the case notes using an Excel-based predesigned data collection form (Additional file 1). Additional linked data on HIV infection status was also sourced from the Paediatric HIV Centre of Excellence (PCOE) within UTH responsible for outpatient care of HIV infected children. Completeness of information on all socio-demographic variables, clinical data including ultimate outcome (death or discharge) as recorded in the registers and legibility of each filled in data collection form were audited at the end of each day to ensure accuracy. Data verification, coding, cleaning and validation were conducted and the cleaned data was then exported to Intercooled Stata version 11 (College Station, Texas, USA) for analysis.

Descriptive statistics were used to summarise age and length of stay on the ward as the data was not normally distributed. Multivariate logistic regression was used to predict the odds of having TB while adjusting for other variables. The variables in the final model included: sex, patient outcome (died/alive), HIV status, types of malnutrition, pneumonia and septicaemia. Trends in TB mortality over the study period were done using Cuzick a non-parametric test for trends. The trend test was done with the assumption that there was no difference in the mortality and discharges during the period under study.

## Results

From 2009 to 2013, a total of 9540 children were admitted with SAM. The median age was 17 (IQR = 22–55 months) and 54% were males. Of these, 151(1.6%, 95% 1.4, 1.9) were diagnosed with TB. The median age at TB diagnosis was 16 months (IQR = 11–24), 56% of TB patients were males. The HIV seroprevalence of children with SAM and TB was 46.5% (n = 66), which was higher than the overall rate of 32.2% in the nutritional unit [21]. TB was more commonly diagnosed among children with Kwashiorkor (47%) compared to marasmic–kwashiorkor (24%) and marasmus (29%). An average of 30 cases of TB per year were diagnosed among children with SAM over the 5-year period reviewed. Twenty-five percent (37/151) of the cases were bacteriologically confirmed. The majority (94%) had pulmonary TB, while TB meningitis, lymphadenitis and disseminated TB were the most common forms of extra-pulmonary TB observed (see Table 1).

**Table 1:** Demographic and clinic-nutritional factors among under-five children with TB and SAM attending UTH in Lusaka, Zambia

Characteristic	TB cases recorded	(%) TB case recorded (n = 151)
Sex		
Male	85	56.3
Female	66	43.7
Age in years		
<1	39	26
1–2	83	55
3–5	29	19
Types of malnutrition		
Marasmic–kwashiorkor	32	24
Marasmus	38	29
Kwashiorkor	62	47
Patient outcome		
Died	84	56
Alive	56	44
HIV status		
Negative	76	53.5
Positive	66	46.5
Patient type		
Bacteriologically confirmed	37	25
Clinically diagnosed	114	27
Type of TB		
Pulmonary TB	142	94
TB adenitis	2	1.3
TB meningitis	5	3.3
Disseminated TB	2	1.3

The case fatality rate among children with SAM and TB was 56% and fluctuated in a non-linear downward trend over the study period of review (see Table 2). No data were available on the TB treatment outcomes of these children because UTH refers all patients to a health facility close to their residence for continued treatment after discharge. On multivariate analysis, children with SAM and TB were 40% more likely to die compared to those without TB. Similarly, children with SAM and HIV infection were twice times more likely to have TB compared to HIV uninfected children. Children with pneumonia were less likely to be diagnosed with TB compared to those without pneumonia (see Table 3).

**Table 2:** Mortality trends in under-five children with SAM and TB attending UTH in Lusaka

Year	2009 n (%)	2010 n (%)	2011 n (%)	2012 n (%)	2013 n (%)	Total n (%)	P value*
Discharged	9 (32)	3 (6)	26 (74)	13 (57)	16 (55)	67 (44)	≤0.0001 for both groups
Died	19 (68)	33 (94)	9 (26)	10 (43)	13 (45)	84 (56)	
Total	28	35	35	23	29	151(100)	

151 is number recorded to have and overall TB prevalence was 1.58 of all admission to the NRU

* P for trend = 0.0001 (tested using cuzick test for trends)

**Table 3:** Multivariate analysis showing factors associated with TB among under-five children with SAM attending UTH, Lusaka

Characteristic	Notified TB		
	N* (% notified TB)	Unadjusted OR (95% CI)	Adjusted OR (95% CI)
Sex			
Male	85 (56.3)	1	1
Female	66 (43.7)	0.9 (0.6–1.2)	0.8 (0.5–1.2)
Age group in months			
<1	39 (26)	1	1
1–2	83 (55)	0.7 (0.5–1.0)	0.8 (0.5–1.3)
3–5	29 (19)	1.0 (0.6–1.6)	1.5 (0.9–2.7)
Patient outcome			
Alive	66 (44%)	1	1
Died	84 (56%)	1.4 (1.0–2.0)	1.4 (1.0–2.1)
HIV status			
Negative	76 (53%)	1	1
Positive	66 (46%)	1.8 (1.3–2.5)	2.3 (1.6–3.3)
Type of malnutrition			
Marasmic–kwashiorkor	32 (24.2)	1	1
Marasmus	38 (28.8%)	0.9 (0.5–1.4)	0.8 (0.5–1.3)
Kwashiorkor	62 (47%)	0.5 (0.3–0.7)	0.5 (0.3–0.8)
Pneumonia			
Without	149 (99)	1	1
With	2 (1)	0.1 (0.04–0.7)	0.1 (0.04–0.6)

n = 151 is the number of children notified to have TB but in the model n = 9540

^*^Tested using multiple logistic regression

## Discussion

This study shows that TB is a contributor to mortality among hospitalised children with severe acute malnutrition. A quarter of the patients were bacteriologically-confirmed using a relatively insensitive method of smear microscopy performed on gastric aspirates. Only 2% of the severely malnourished children were diagnosed with TB in this high HIV and TB prevalence setting suggesting under-detection.

This study had limitations. Retrospective studies are inherently prone to biases. We were limited on the amount of information and quality of data we could collect on the subjects based on the available records. Record keeping in most poor resource settings is challenging and this may have resulted in missing data or under-reporting of TB cases. Additionally we were not able to confirm the exact causes of death as autopsies were not routinely performed in this setting [22]. We were also unable to obtain post-discharge TB treatment outcomes and therefore cannot ascertain the impact of SAM on TB treatment outcomes.

Overall the mortality rate due to TB was high compared to overall mortality in the nutritional unit [21, 23]. Data is lacking on mortality in severely malnourished children with TB in the African setting. However, Chisti et al. [24] reported no in-hospital mortality in Bangladesh but high post-discharge mortality in severely malnourished children with TB. Late presentation, presence of HIV infection and young age are associated with increased mortality due to TB [25, 26].

Relying on smear microscopy performed on gastric aspirates, our study finding of 25% bacteriologic confirmation was comparatively similar to that seen in Asia but higher than in other high TB and HIV endemic settings of Malawi and South Africa [27, 28]. Using more sensitive Xpert MTB RIF and mycobacterial culture contributed to high confirmation in Asia but was of no additional value in severely malnourished children in Malawi. Our findings may have under-estimated the true burden of TB among children with SAM. Diagnosis of TB in children is traditionally challenging especially in the presence of SAM owing to the overlap of clinical features between TB and malnutrition. The high HIV co-infection may have compounded the diagnosis of TB given the non-specificity of clinical features in the presence of HIV and SAM [29]. We also found a lower likelihood of TB among cases with pneumonia inconsistent with the known risk of TB among children with respiratory infections and particularly with malnutrition [30, 31]. Two autopsy studies performed at this institution have consistently found TB as an important cause of death among children dying from respiratory disease and that malnutrition is a frequent comorbidity [22, 32]. This finding coupled with fewer cases of extra-pulmonary TB, in this younger cohort typically prone to primary TB disease are all pointers to under-detection.

The occurrence of TB alongside SAM and HIV calls for improved case detection to establish the true burden of TB disease and prevent the high TB-related mortality. The use of improved and more sensitive diagnostic approaches such as Xpert MTB/RIF or Xpert MTB/RIF Ultra, and mycobacterial cultures as well as the use of alternative specimen collection methods to gastric lavage such as sputum induction, nasopharyngeal aspirates and stools should be explored in children with SAM. Integrative care approaches that optimise inclusion of TB screening and prevention within existing nutritional rehabilitation, maternal and child health services and other HIV service areas are required for improved case detection [30, 33].

## Conclusion

Tuberculosis contributes significantly to mortality among children with SAM in high TB and HIV prevalence settings. The comorbidity of HIV alongside TB and SAM justifies the need for an integrative approach for optimal TB management within existing mother and child health interventions. Innovative approaches for improved TB case finding, contact screening and provision of isoniazid preventive therapy are required in children with SAM.

## Additional file

**Additional file 1.** The dataset supporting the conclusions of this article

## Abbreviations

HIV: human immunodeficiency virus
OR: odds ratios
OPT: outpatient therapeutic care Programs
NRU: Nutrition rehabilitation unit
PMTCT: prevention of mother to child transmission
RUFT: ready to use therapeutic food
SAM: severe acute malnutrition
SD: standard deviation
TB: tuberculosis
UTH: University Teaching Hospital
WHO: World Health Organization
WHZ: weight for height Z-score

## References

[1] Jenkins HE (2016). Global burden of childhood tuberculosis. Pneumonia.

[2] WHO (2016). Global tuberculosis report.

[3] Ahmed T, Hossain M, Sanin KI (2012). Global burden of maternal and child undernutrition and micronutrient deficiencies. Ann Nutr Metab.

[4] Levels and trends in child malnutrition: UNICEF/WHO/World-Bank joint child malnutrition estimates—Key findings of the 2016 edition. (http://www.who.int/nutgrowthdb/jme_brochure2016.pdf).

[5] WHO (2013). Guideline: nutritional care and support for patients with tuberculosis.

[6] Nelson LJ, Wells CD (2004). Global epidemiology of childhood tuberculosis. Int J Tuberc Lung Dis.

[7] Rieder HL. Epidemiologic basis of tuberculosis control. Paris: International Union Against Tuberculosis and Lung Disease; 1999. pp. 1–164.

[8] Jaganath D, Mupere E (2012). Childhood tuberculosis and malnutrition. J Infect Dis.

[9] Vijayakumar M, Bhaskaram P, Hemalatha P (1990). Malnutrition and childhood tuberculosis. J Trop Pediatr.

[10] Kapata N, Chanda-Kapata P, Ngosa W, Metitiri M, Klinkenberg E, Kalisvaart N, Sunkutu V, Shibemba A, Chabala C, Chongwe G (2016). The prevalence of tuberculosis in Zambia: results from the First National TB Prevalence Survey, 2013–2014. PLoS ONE.

[11] WHO (2016). Global tuberculosis report 2016.

[12] MOH. National TB/Leprosy program 2016 TB notification data. Lusaka: Department of Public Health; 2017.

[13] MOH. Zambia population-based HIV impact assessment (Zamphia) 2015–2016, summary sheet: preliminary findings. Lusaka: Ministry of Health; 2016.

[14] Bates M, O’Grady J, Maeurer M, Tembo J, Chilukutu L, Chabala C, Kasonde R, Mulota P, Mzyece J, Chomba M (2013). Assessment of the Xpert MTB/RIF assay for diagnosis of tuberculosis with gastric lavage aspirates in children in sub-Saharan Africa: a prospective descriptive study. Lancet Infect Dis.

[15] Chabala C, Somwe S, Kapata N, Chongwe G, Jumbe-Marsden E. Childhood tuberculosis at the main referral hospital in Lusaka, Zambia—A five year review of registered child TB cases. In: 43rd Union conference on Lung Health. vol. PC-661-15. Kuala Lumpur; 2012.

[16] MOH. Zambia National Health Strategic Plan 2011–2015: towards attainment of health related millennium development goals and other national health priorities in a clean, caring and competent environment. Lusaka: Ministry of Health; 2010.

[17] CSO, MoH, TDRC. Zambia demograpics and health survey 2007. Calverton, Maryland; 2009.

[18] CSO M, ICF. Zambia Demographic and Health Survey 2013–14. 2014, Rockville, Maryland: Central Statistical Office, Ministry of Health, and ICF International.

[19] MoH, UNICEF: Zambian guidelines for antiretroviral therapy of HIV infection in infants and children: towards universal access. Lusaka: Recommendations for a publlic health approach. Ministry of Health; 2007.

[20] WHO. Guideline update: Technical aspects of the management of severe acute malnutrition in infants and children. In: Edited by Development DoNfHa. Geneva: World Health Organisation; 2013.

[21] Munthali T, Jacobs C, Sitali L, Dambe R, Michelo C (2015). Mortality and morbidity patterns in under-five children with severe acute malnutrition (SAM) in Zambia: a five-year retrospective review of hospital-based records (2009–2013). Arch Public Health.

[22] Bates M, Shibemba A, Mudenda V, Chimoga C, Tembo J, Kabwe M, Chilufya M, Hoelscher M, Maeurer M, Sinyangwe S (2016). Burden of respiratory tract infections at post mortem in Zambian children. BMC Med.

[23] Irena AH, Mwambazi M, Mulenga V (2011). Diarrhea is a major killer of children with severe acute malnutrition admitted to inpatient set-up in Lusaka, Zambia. Nutr J.

[24] Chisti MJ, Graham SM, Duke T, Ahmed T, Ashraf H, Faruque AS, La Vincente S, Banu S, Raqib R, Salam MA (2014). A prospective study of the prevalence of tuberculosis and bacteraemia in Bangladeshi children with severe malnutrition and pneumonia including an evaluation of Xpert MTB/RIF assay. PLoS ONE.

[25] Jenkins Helen E, Yuen Courtney M, Rodriguez Carly A, Nathavitharana Ruvandhi R, McLaughlin Megan M, Donald Peter, Marais Ben J, Becerra Mercedes C (2017). Mortality in children diagnosed with tuberculosis: a systematic review and meta-analysis. The Lancet Infectious Diseases.

[26] Fergusson P, Tomkins A (2009). HIV prevalence and mortality among children undergoing treatment for severe acute malnutrition in sub-Saharan Africa: a systematic review and meta-analysis. Trans R Soc Trop Med Hyg.

[27] LaCourse SM, Chester FM, Preidis G, McCrary LM, Arscott-Mills T, Maliwichi M, James G, McCollum ED, Hosseinipour MC (2014). Use of Xpert for the diagnosis of pulmonary tuberculosis in severely malnourished hospitalized Malawian children. Pediatr Infect Dis J.

[28] De Maayer T, Saloojee H (2011). Clinical outcomes of severe malnutrition in a high tuberculosis and HIV setting. Arch Dis Child.

[29] Marais BJ, Rabie H, Cotton MF (2011). TB and HIV in children—advances in prevention and management. Paediatr Respir Rev.

[30] Graham SM, Sismanidis C, Menzies HJ, Marais BJ, Detjen AK, Black RE (2014). Importance of tuberculosis control to address child survival. Lancet.

[31] Oliwa JN, Karumbi JM, Marais BJ, Madhi SA, Graham SM (2015). Tuberculosis as a cause or comorbidity of childhood pneumonia in tuberculosis-endemic areas: a systematic review. Lancet Respir Med.

[32] Chintu C, Mudenda V, Lucas S, Nunn A, Lishimpi K, Maswahu D, Kasolo F, Mwaba P, Bhat G, Terunuma H (2002). Lung diseases at necropsy in African children dying from respiratory illnesses: a descriptive necropsy study. Lancet.

[33] Detjen A, Gnanashanmugam D, Talens A. A framework for integrating childhood tuberculosis into community-based child health care. 2013.

